# Generation of a Ym1 deficient mouse utilising CRISPR-Cas9 in CB6 embryos

**DOI:** 10.1007/s11248-025-00455-8

**Published:** 2025-09-25

**Authors:** J. E. Parkinson, G. E. Baldwin, P. H. Papotto, N. E. Humphreys, A. J. Day, A. D. Adamson, J. E. Allen, T. E. Sutherland

**Affiliations:** 1 Lydia Becker Institute of Immunology and Inflammation, School of Biological Sciences, Faculty of Biology, Medicine and Health, University of Manchester, Manchester, M139PT UK; 2 Manchester Cell Matrix Centre, School of Biological Sciences, Faculty of Biology, Medicine and Health, University of Manchester, Manchester, M139PT UK; 3 Genome Editing Unit, School of Biological Sciences, Faculty of Biology, Medicine and Health, University of Manchester, Manchester, Manchester, M139PT UK; 4 European Molecular Biology Laboratory (EMBL), Epigenetics & Neurobiology Unit, Rome, Italy; 5 Institute of Medical Sciences, School of Medicine, Medical Sciences and Dentistry, University of Aberdeen, Aberdeen, AB252ZD UK

**Keywords:** CLPs, Gene duplication, Mouse model, CRISPR, Mixed background

## Abstract

Chitinase-like proteins (CLPs) are of wide interest due to their significant roles during both homeostatic and pathological processes. Human CLPs such as YKL-40 have been proposed as biomarkers of disease severity in many conditions. Murine CLPs Brp39, Ym1, and Ym2 are similarly upregulated in multiple mouse models of pathology. Investigation of Ym1 and Ym2 is hampered by recent gene duplication events on the C57BL/6, but not BALB/c, background leading to complexity in the genomic locus. Here, we have generated a Ym1 deficient mouse using a novel CRISPR-Cas9 targeting approach involving CB6 (C57BL/6 X BALB/c) mixed background embryos. Validation using flow cytometry, ELISA, and immunofluorescence confirmed no expression of mature Ym1 protein. Additionally, expression of related genes including *Chia, Chil1,* and *Chil4* were not altered in Ym1-deficent animals. This new transgenic mouse line will be key for future investigations of CLP functions and the utilised approach to genetic manipulation may provide a useful strategy for other genes which show differences in copy number between inbred mouse strains.

**Supplementary Information:**

The online version contains supplementary material available at 10.1007/s11248-025-00455-8.

## Introduction

Chitinase-like proteins (CLPs) are expressed in a range of organisms including mammals (Bussink et al. 2007), insects (Arakane and Muthukrishnan 2010; Kucerova et al. 2015; Zhang et al. 2011), parasites (Bando et al. 2024; He et al. 2017), and plants (Ho and Ng, n.d.; Kesari et al. 2015; Xu et al. 2023). They are part of the glycoside hydrolase 18 family and have evolved from gene duplication events of their ancestral precursors, the active chitinases (Bussink et al. 2007; Hussain and Wilson 2013). Chitinases catalyse the breakdown of chitin, the second most abundant natural polymer on earth after cellulose. Chitin is a structural and protective molecule found in Fungi and several phyla of Animalia; notably Nematoda and Arthropoda. Chitinases evolved not only to act as host defence molecules against chitin-containing pathogens but also function in degrading inhaled chitin from environmental sources (Van Dyken et al. 2017). In addition, humans today are widely exposed to chitin from its use in industry, medicine, and biotechnology. Whilst some CLPs can still bind chitin, they have lost chitin degrading ability (Kesari et al. 2015). Despite this, CLPs maintain an expression pattern broadly associated with conditions of type-2 inflammation such as nematode infection and asthma (Elias et al. 2005; Sutherland et al. 2014; Kesari et al. 2015; Sutherland 2018; Liu et al. 2019; Declercq et al. 2023).

The CLP family contains a diverse array of genes across many species and these molecules often show high sequence identity (Table 1). The different members are thought to have evolved from birth and death evolution under strong purifying selection (Nei and Rooney 2005). This has resulted in the independent evolution of species-specific genes, with orthologs generally being more closely related than paralogs (Bussink et al. 2007). Humans have two main CLPs, YKL-40 (*CHI3L1*), and YKL-39 (*CHI3L2*). YKL-40 in particular has repeatedly been suggested as a biomarker of disease severity across many conditions (Craig-Schapiro et al. 2010; Johansen et al. 2006; Wang et al. 2008; Rathcke et al. 2006; Zhao et al. 2020). Notably increased YKL-40 protein levels have been reported in the serum in people with certain asthma endotypes (Liu et al. 2019; Konradsen et al. 2013) and genome wide association studies have highlighted *CHI3L1* as a risk gene for asthma (Gomez et al. 2015). Despite these observations, the exact function of YKL-40 is still not well understood.

**Table 1:** Murine and Human chitinase-like proteins

Human		Mouse	
Gene	Protein	Gene	Protein
**CHIT1**	**Chitotriosidase**	**Chit1**	**Chitotriosidase**
CHI3L1	YKL-40	Chil1	Brp39
CHI3L2	YKL-39		
**CHIA**	**AMCase**	**Chia**	**AMCase**
OVGP1	Oviductin	Ovgp1	Oviductin
		Chil3	Ym1
		Chil4	Ym2

Active chitinases are shown in bold and orthologous genes are listed in the same rows. Chitinase-like proteins are listed under the gene they originally evolved from

In contrast to humans, mice have three coding CLPs, Brp39 (*Chil1*), Ym1 (*Chil3*), and Ym2 (*Chil4*) which have also been implicated in a plethora of diseases (Kang et al. 2022). Ym1 and Ym2 in particular have very high sequence identity at the exonic and protein level. This has hindered study of these molecules due to a lack of tools able differentiate between these two molecules in situ. We recently reported distinct expression patterns of Ym1 and Ym2 in the mouse lung (Parkinson et al. 2021), highlighting potential differences in their respective function. These CLPs are also under purifying selection, suggesting they have necessary functions in health and disease (Bussink et al. 2007; Okawa et al. 2023). Although *Chil3* and *Chil4* were identified around thirty years ago (Jin et al. 1998), their biological functions, like their human counterparts, remain elusive.

The CRISPR-Cas9 editing system was initially discovered in bacteria and archaea where it functions to protect against invasive viral and plasmid nuclei acids (Jansen et al. 2002; Wiedenheft et al. 2012). Since then it has been adapted to work as a DNA editing system in eukaryotic cells (Jinek et al. 2012; Cong et al. 2013; Mali et al. 2013). CRISPR-Cas9 has now become the gold standard for the generation of genetically altered mice (Seruggia and Montoliu 2014). In this study we sought to generate a Ym1-deficient mouse strain, due to genetic complexities this ultimately required the use of CRISPR-Cas9 mediated deletion in a hybrid background strain. This new mouse line will enable specific investigation of the role of Ym1 in disease aetiology and pathogenesis.

## Results

### Chil3 and Chil4 encode for homologous CLPs

**Fig. 1:**
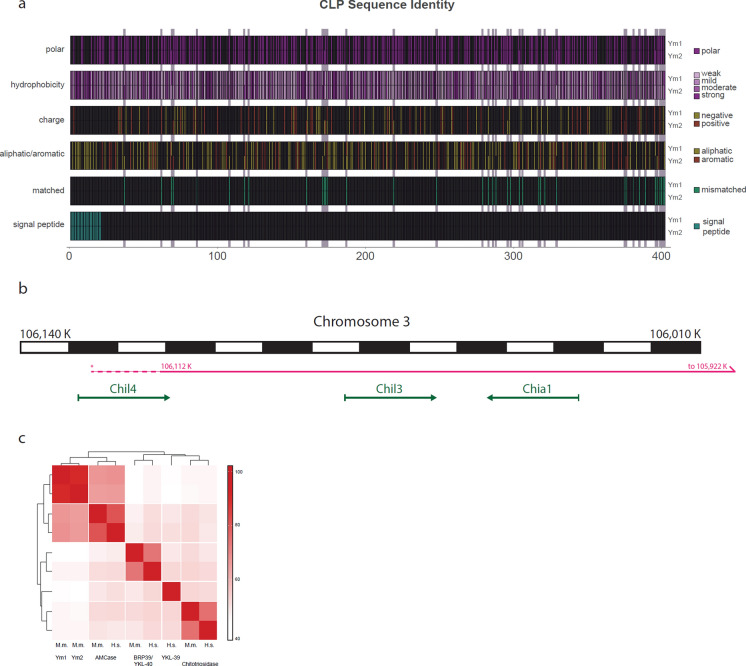
The murine chitinase like proteins (CLPs) Ym1 and Ym2 have high sequence identity (91% for the mature proteins). **a** Schematic showing the sequence positions where amino acids are different between the two proteins (grey bars) and associated differences in biochemical properties. The genes that encode Ym1 (*Chil3*) and Ym2 (*Chil4*) are also located immediately adjacent to each other on chromosome 3, **b** Schematic showing the location of the *Chil3* and *Chil4* genes on chromosome 3 in relation to *Chia,* which encodes for AMCase. Black and white alternating blocks represent 10 kb of DNA, Solid magenta line shows the duplication suggested by data from Graubert et al. 2007. Dashed magenta line shows an extension that is supported by the data within this manuscript. Relevant genes and their orientation on chromosome 3 are shown by green arrows. **c**) Heatmap showing the amino acid sequence identity between key members of the glycoside hydrolase 18 family in mouse and human. Scale shows amino acid sequence identity in percent. (M.m = Mus. musculus and H.s = Homo. Sapiens)

Chitinase-like proteins show high sequence identity across, and especially within, species. In particular, Ym1 (*Chil3*) and Ym2 (*Chil4*) have > 90% sequence identity at the protein, ribonucleic acid (RNA), and deoxyribonucleic acid (DNA) level. While these proteins share a similar 3D structure (Sun et al. 2001; Heyndrickx et al. 2023), differences in the amino acid sequence will lead to some alteration of their biochemical properties e.g. due to charge distribution (Fig. 1a). However, the potential impact of these differences is not well understood. *Chil3* and *Chil4* have arisen from recent gene duplication events (Bussink et al. 2007; Funkhouser and Aronson 2007) and are located immediately adjacent to each other on chromosome three (Fig. 1b). Within the human and mouse Glycoside Hydrolase Family 18 (GH18) genes, *Chil3* and *Chil4* uniquely show the highest sequence identity to each other. This is in comparison to other GH18 members which show greater identity with their orthologs, rather than associated paralogs. (Fig. 1c).

### Strategy for generating a Chil3 floxed mice

**Fig. 2:**
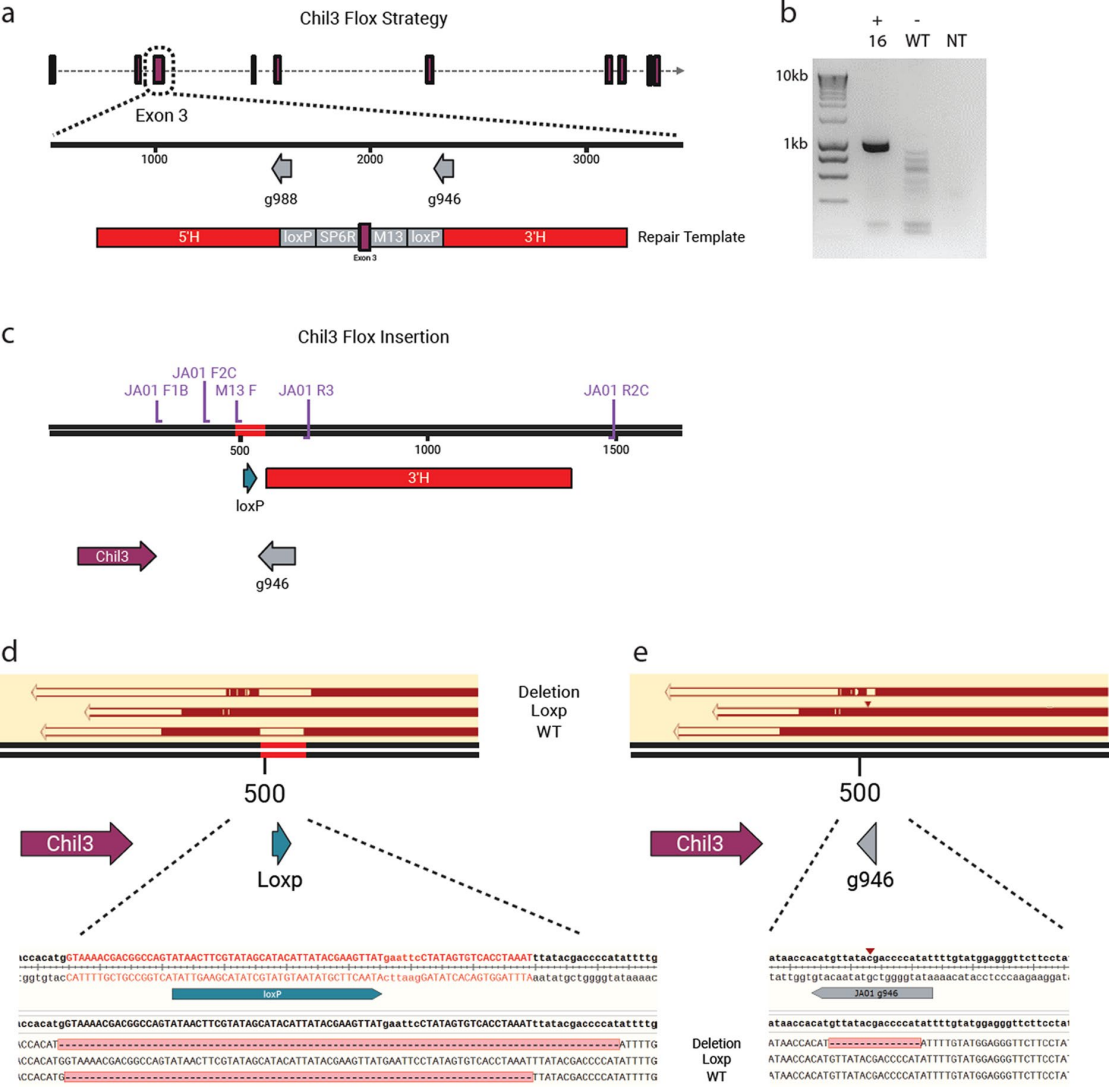
Genetic targeting strategy for generating a *Chil3* floxed mouse. **a** Schematic showing the homology directed repair template including 3’ and 5’ homology arms and relative location to the *Chil3* gene. **b** Genotyping results from the M13F and JA01 R2C primer sequences showing amplification of a 1 kb product in pup 16 but not in C57BL/6 control DNA (WT) or non-template control (NT). **c**) Projected results from the methodology in a) and b) demonstrating the inclusion of an intronic LoxP site and flanking 3’ homology arm as well as respective primer binding sites. F1B/R2C PCR amplicons from pup 16 were blunt end ligated into plasmids followed by transfection into competent cells. These were then grown up and transformed colonies were miniprepped and Sanger sequenced using the M13R primer. **d**-**e** Sanger sequencing results of pup 16 products aligned to the predicted HDR knock in sequence **d** and the Chil3 reference sequence **e** showing the presence of three different alleles including the LoxP insertion, 15 bp deletion, and wild type sequence. Note the small red triangle indicates an insertion

Targeted gene knockout using CRISPR-Cas9 mediated deletion is a classic approach to study gene function. However, the *Chil3* gene is a particularly challenging target due to its high sequence identity to *Chil4* (Fig. 1) (Hussain and Wilson 2013). Design of specific exonic *Chil3* single guide RNAs (sgRNA) proved difficult. However, intronic sequences were sufficiently different between *Chil3* and *Chil4* to allow generation of specific single guide (sg)-RNAs (g988 and g946) (Fig. 2a). Alongside this approach, we utilised a flanking LoxP strategy to allow spatiotemporal control of protein expression and mitigate against potential lethality. Specific sgRNAs were synthesised and cloned into a complementary DNA repair template compromising a Homology-loxP-SP6R-Exon3-M13F-loxP-Homology sequence (Fig. 2a). These sgRNA, Cas9, and DNA donor were then co-delivered to C57BL/6J mouse embryos, were transferred to pseudopregnant CD1 recipients.

Screening of the initial F0 pups revealed low editing efficiency, but two mice showed the presence of a single LoxP integration, a common outcome of such experiments (Gurumurthy et al. 2019). Pup 16 had integrated the 3’ LoxP site, as determined by polymerase chain reaction (PCR) (Fig. 2b) using the M13F primer sequence integrated as part of the donor template, and a reverse primer targeted to the flanking homology arm (JA01 R2C) (Fig. 2c). This LoxP site integration was then confirmed by Sanger sequencing of an encompassing PCR amplicon (JA01 F1B and R2C) (Fig. 2d and e). This sequencing revealed three distinct products: the WT allele, LoxP insert, and a deletion of 15 bases, suggesting potential mosaicism in the F0 animal (Fig. 2d and e). An additional pup had integrated the 5’ LoxP, as determined by similar assays targeted to the 5’ end of the integration site (data not shown). We established a colony of mice bred from Pup 16, with a view to harvesting embryos from mice homozygous for the 3’ LoxP. This would allow us to introduce the 5’ LoxP on this background using CRISPR and a single-stranded oligodeoxynucleotide repair template. Pup 16 was crossed with a WT C57BL/6J male and germline transmission confirmed using the same flanking PCR as previous (Fig. 2c). Heterozygote mice with the LoxP site were then interbred to establish a homozygote colony.

### Identification of coinherited alleles and gene duplication of the CLP locus

**Fig. 3:**
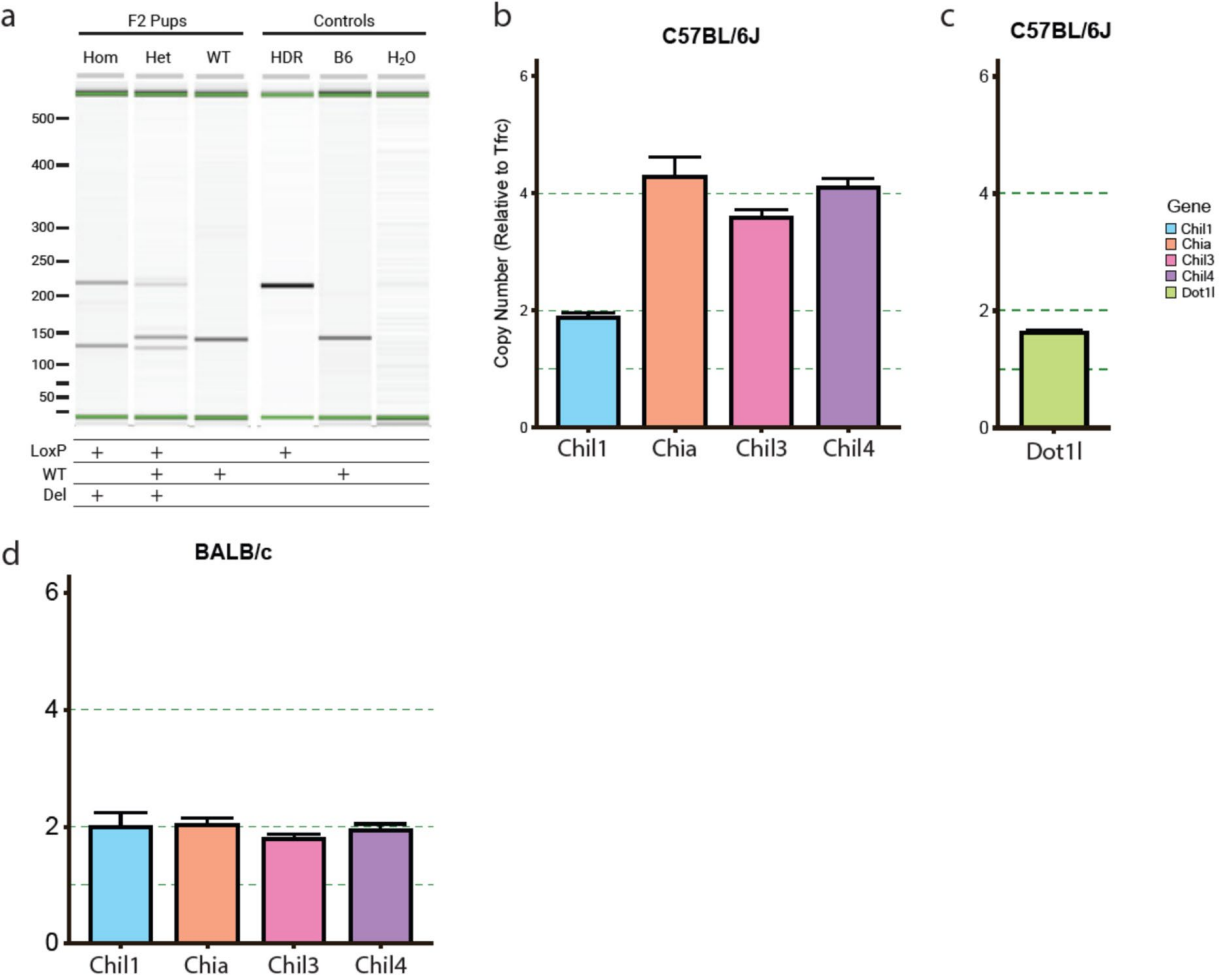
PCR Confirmation of the F2 generation of the *Chil3* floxed mice. **a** Genotyping results using a Qiaxcel automated gel system. Pups were genotyped for the presence of the LoxP or WT sequence using the JA01 and F1C/R3 primers. Coinheritance of the LoxP site and the 15 bp deletion (see Fig. 2) was demonstrated in the homozygote mice, which showed no presence of the WT allele. WT mice showed no presence of the LoxP insert or the 15 bp deletion while Het animals showed the presence of all three bands. The identity of these bands was confirmed using spiked HDR template (HDR) and WT C57BL/6 J (B6) control sequences in addition to a negative non-template control (H_2_O) shown on the right three lanes of the gel. **b** Confirmation of the CLP gene duplication using droplet digital PCR showing the copy number of *Chil3* and *Chil4* C57BL/6 J mice in reference to a control gene (*Tfrc*) with a copy number of two. **c**) ddPCR confirmation of equivalent copy number between *Dot1l* and *Tfrc* in C57BL/6 J mice **d**) Validation that BALB/c mice do not have the duplication of the CLP locus via the same ddPCR assay in b)

The F2 generation of pups were screened using a PCR reaction (JA01 F2C/R3) designed to amplify smaller products, 136 bp (WT) or 211 bp (LoxP). This reaction allowed us to identify the LoxP site more easily on a Qiaxcel automated gel system. However, when analysed an unexpected smaller amplicon was also observed in the heterozygous (Het) and homozygous (Hom) pups (Fig. 3a). The additional smaller amplicon corresponded to the previously identified CRISPR induced deletion (Fig. 2) and co-segregated with the LoxP site (Fig. 3a). The co-segregation of both a Homology-directed repair (HDR) knock in and a non-homologous end joining (NHEJ) deletion in the same region led us to hypothesise that this region on chromosome 3 was duplicated in the C57BL/6 background strain. To test this, genomic DNA was harvested from WT C57BL/6 J mice and analysed by droplet digital PCR (ddPCR) using probes designed against *Chil3* and *Chil4* alongside a reference *Tfrc* probe (located on chromosome 16). This assay showed a copy number duplication (4) in the C57BL/6 strain compared to the reference (2) (Fig. 3b). The *Tfrc* reference gene was also cross validated to another gene *Dot1l* which showed the same copy number (2) (Fig. 3c). The co-segregation of the two editing events could be explained by simultaneous targeting of the duplicated *Chil3* gRNA binding sites which exist on the same allele. To this end we searched the literature for studies characterising regions of copy number variation in the C57BL/6 J genome. We found a report on copy number variations in the mouse genome which identified a duplication event in the CLP locus across several inbred mouse strains including the C57BL/6 substrains (Graubert et al. 2007). Notably, this duplication was not identified in the BALB/c strain, among others, which we validated using our ddPCR assay on WT BALB/c animals (Fig. 3d). The presence of this gene duplication explained the complications with CRISPR targeting in the C57BL/6 strain.

### Utilising mixed background CB6 mice to generate a Chil3 deficient mouse

**Fig. 4:**
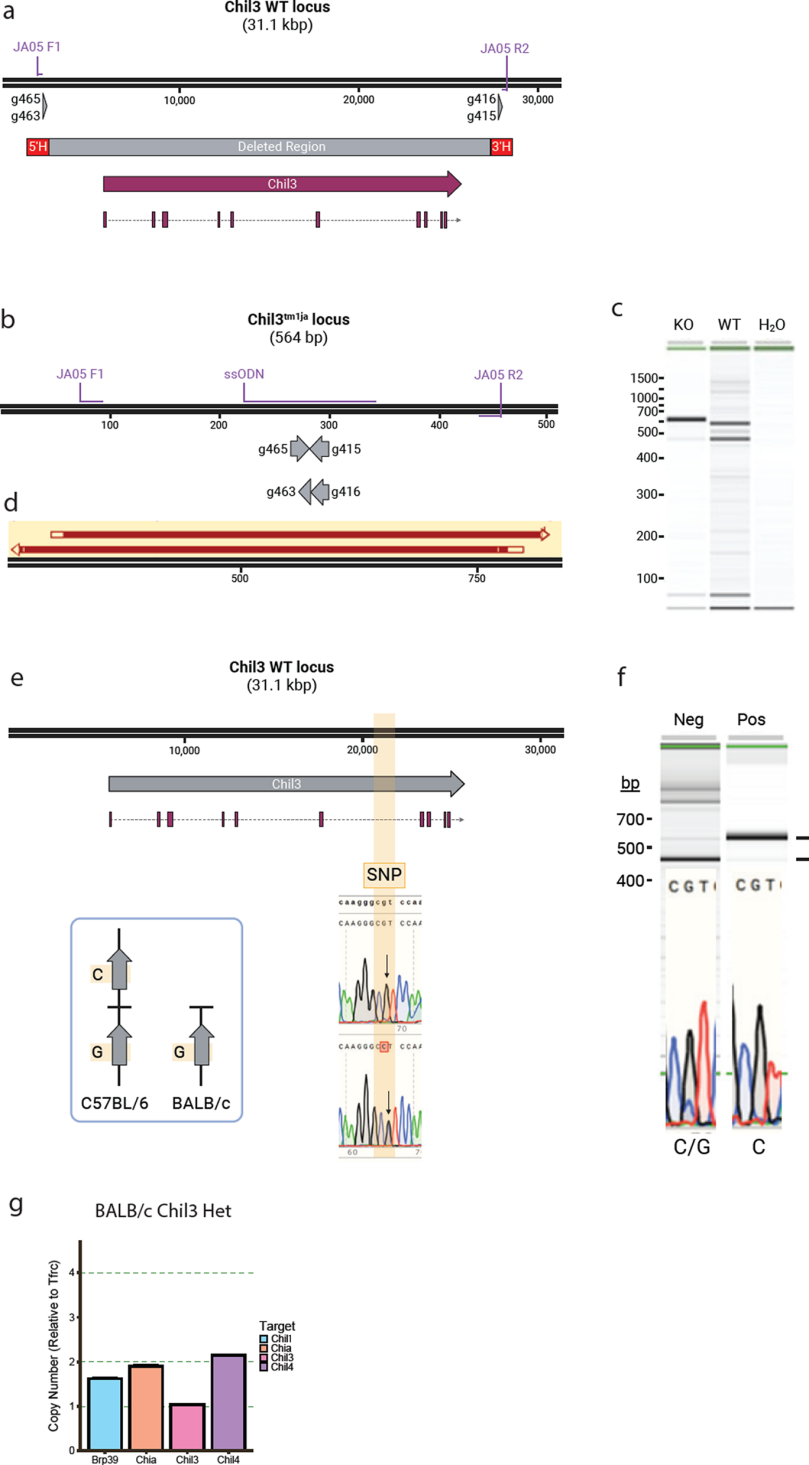
Strategy for generating a *Chil3*-deficient mouse. **a** Schematic showing the guide RNAs and the expected deletion used in BALB/c and CB6 embryos. **b** Schematic showing the expected deletion and respective oligo binding locations. **c** PCR results using JA05 F1 and JA05 R2 amplifying a 564 bp band in the KO animals, but not in the WT controls. **d** Sanger sequencing confirming the sequence of the 564 bp product in c) aligned with the predicted KO sequence in b). **e** Schematic showing the location of strain specific SNPs in the *Chil3* gene with sanger sequencing confirming the sequence of these SNPs between C57BL/6 and BALB/c mice and the differences between gene copies in B6 animals. **f** Sanger sequencing showing the presence of a pure C read in the SNP of a pup containing the deletion shown in a) and a mixed G/C read in a littermate control without the mutation. **g**) Droplet digital PCR showing the copy number of *Chia1*, *Chil1*, *Chil3*, and *Chil4* in heterozygous Chil3-deficient offspring. Showing that there is a copy number of one for Chil3 indicating there has been a deletion of this gene, but with no change to the copy number of *Chil1*, *Chia1*, or *Chil4*

To avoid targeting issues associated with the duplication of the CLP locus in C57BL/6 J mice we utilised an alternative knockout strategy. We planned to delete the entire *Chil3* gene in BALB/c mice which lack the CLP duplication event (Fig. 3d) but remain a viable model for immunology research. Using BALB/c mice enabled the leverage of intergenic regions for specific sgRNA design, whilst also allowing design of a single-stranded DNA repair template for precise excision of the full gene (Fig. 4a). Disappointingly, standard superovulation and embryo harvesting techniques in BALB/c mice generated poor quality embryos with a low survival rate (64%) and few viable pregnancies. Only 3 pups were born from a total of 346 embryos injected (0.9%), none with the correct deletion (Table 2). To overcome this, we hypothesised that using CB6 (C57BL/6 × BALB/c) embryos would give us a single *Chil3* BALB/c allele to target, alongside the better embryo characteristics associated with C57BL/6 animals. Embryo retrieval and survival rates were improved compared to pure BALB/c embryos (81%). However, only a modest increase in pup numbers was observed, with 3 pups born from a total of 177 embryos injected (1.7%) (Table 2), (Table 3). These pups were genotyped using primers that would amplify deletion events (Fig. 4b) but give no product in unedited mice (JA05 F1/R1) (Fig. 4a, b and Table 4). A single CB6 pup showed a band at the predicted size of 564 bp (Fig. 4b, c and d), which Sanger sequencing revealed to be a precise, HDR mediated deletion of the *Chil3* gene (Fig. 4d). This founder was then backcrossed with a BALB/c mouse in order to confirm germline transmission of the deletion (Sup Fig. 1a).

**Table 2:** Embryo survival rates

Strain	Total Injected	Embryo Survived (survival rate %)	Viable Pregnancy	Pups born (% of embryos injected)	Gene edited mice
C57BL/6	745	719 (97)	-	73 (9.8)	5
BALB/c	346	222 (64)	1/10	3 (0.9)	0
CB6	177	144 (81)	1/6	3 (1.7)	1

**Table 3:** Mouse strains

Item	Code	
BALB/c^OlaHsd^	MGI:3,047,778	
C57BL/6J^OlaHsd^	MGI:2,164,189

**Table 4:** Primers used for genotyping and sequencing edited pups

Name	Sequence	
JA01 F1	tcccatgtcaataccatacagaa	
M13 R	caggaaacagctatgac	
JA01 F1B	tctcattgcttcctaaagtgtatgt	
JA01 F2C	tcaatcatgtccttgattctgcat	
M13 F	gtaaaacgacggccagt	
JA01 R2C	tccttgggacactgttctcaaaa	
JA01 R3	ggtcaaaccacagaatgaacct	
JA05 F1	acacccaggcaccatagaaa	
JA05 R1	tcagccattcaaggttcctct	
JA05 SNP F2	ggttctggttcaactggtggt	
JA05 SNP R2	tctcagccatttgatattacgca	
JA05 SNP R	tgggtgccattttgtcctactg	

As the mutation could have targeted one of the duplicated C57BL/6 alleles, or the desired BALB/c allele, we needed to determine which copy of the *Chil3* gene was deleted in the founder. Potential single nucleotide polymorphisms (SNPs) between the strains that could be exploited to determine which allele had been deleted were identified from publicly available databases (http://www.informatics.jax.org/snp). This region of the mouse genome contained only one candidate SNP for this assay, an intronic base predicted to be a C nucleotide in C57BL/6 and a G nucleotide in BALB/c (Fig. 4e). This sequence was amplified (JA05 SNP F2/R2) from both C57BL/6 and BALB/c genomic DNA and Sanger sequenced (JA05 SNP R) to confirm the webtool prediction. The BALB/c SNP showed as a G nucleotide, but an overlapping peak indicated both a G and C nucleotide in the C57BL/6 strain (Fig. 4e). Nevertheless, the SNP could still be exploited for genotyping. If the BALB/c *Chil3* allele was deleted in the founder, crossing with WT BALB/c mice would give a pure G read in this amplicon in F1 pups harbouring the deletion, whereas littermates without the deletion would transmit the C57BL/6 allele giving a mixed GC read (Sup Fig. 1b). A pure G read was observed in F1 litters harbouring the deletion, indicating deletion of the desired BALB/c *Chil3* gene in the founder compared to WT littermate DNA which showed a mixed G/C read (Fig. 4e). Final quantification of *Chil3* copy number was confirmed via ddPCR. As expected *Chil3* showed a copy number of one, whilst *Chil1*, *Chia*, and *Chil4* all showed the normal copy number of two. This aligned with the inheritance of the edited BALB/c CLP locus (with a *Chil3* deletion) alongside a copy of the WT BALB/c allele (Fig. 4g). The loss of *Chil3* copy number between WT BALB/c DNA and the F1 animals resulted due to the loss of the entire gene which removed the binding sites for the primers in our ddPCR assay (Fig. 4b and g).

### Speed congenics allows rapid backcrossing of CB6 pups to common lab strains

**Fig. 5:**
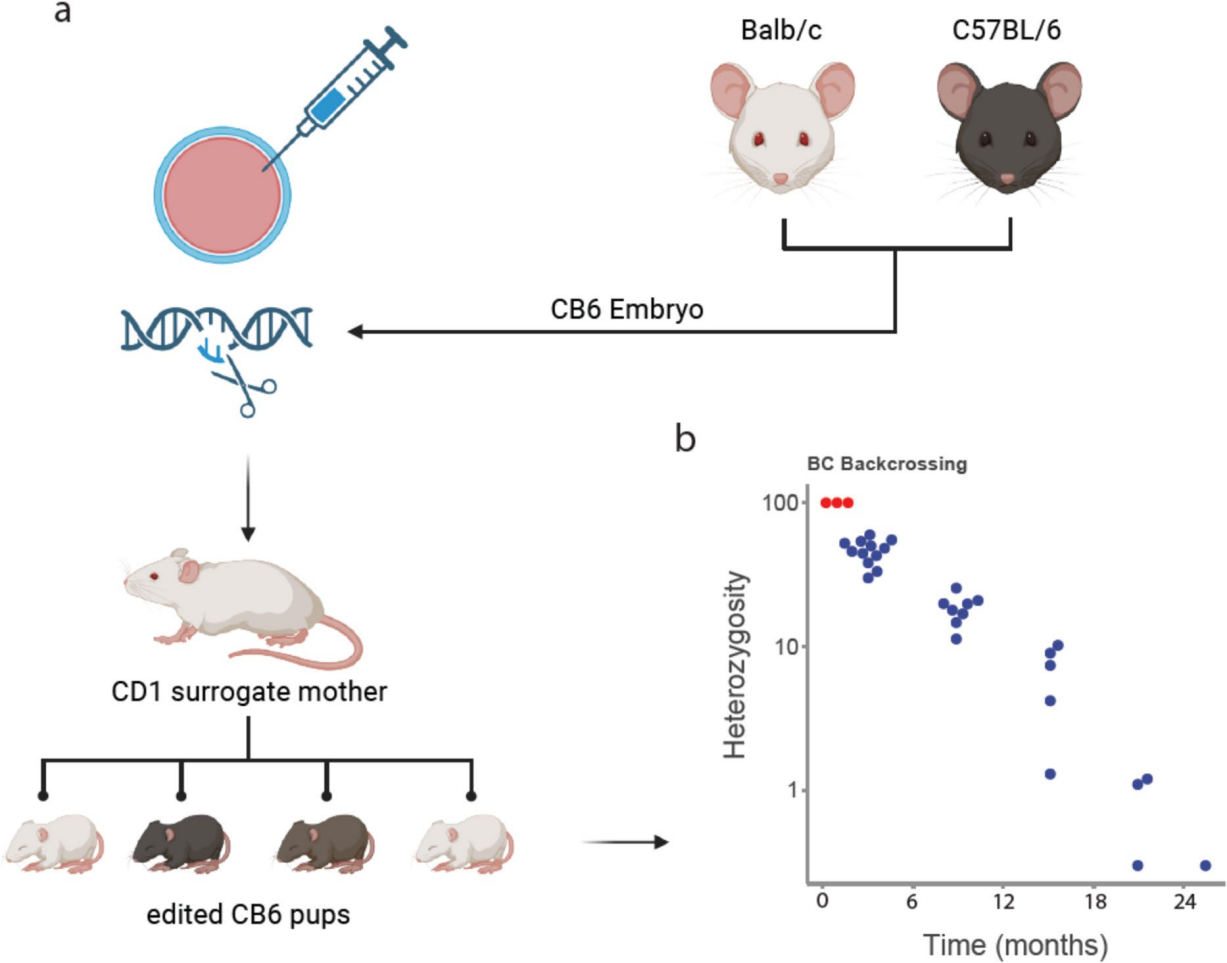
To facilitate further characterisation and analysis the CB6 pups carrying the *Chil3* deletion were backcrossed onto a BALB/c background. **a** strategy used to generate CB6 embryos for microinjection. **b** Speed congenics using the Transnetyx genetic monitoring service was then used to quantify the heterozygosity across each pup’s genome and the animals with the lowest heterozygosity was used for future breeding allowing completed transfer to the BALB/c background in five generations

There are variances in responsiveness to certain pathogenic challenges between different inbred mouse strains (Wakelin 1993; Sacks and Noben-Trauth 2002; Cliffe and Grencis 2004; Maizels et al. 2012; Grencis 2015; Finlay and Allen 2020; Parkinson et al. 2021). To facilitate investigation into this and to provide a homogenous background on which to analyse *Chil3*-deficient animals, heterozygote mice were backcrossed to wildtype C57BL/6 and BALB/c mice. Speed congenics utilises a panel of strain specific SNPs to identify the optimal animal to use for breeding (Fig. 5a) (Transnetyx speed congenic service) (Fahey et al. 2013; ‘The Importance of Monitoring Genetic Background in Genetically Modified Mouse Colonies’, Transnetyx White Paper; ‘Speed Congenics Efficiency, Economy, Stability, Consistency, and Transparency’, Transnetyx White Paper). This allowed rapid stabilisation onto the BALB/c background, resulting in a fully backcrossed strain in five generations (Fig. 5b).

### Chil3-deficient animals do not express Ym1 but show no change in related CLPs

**Fig. 6:**
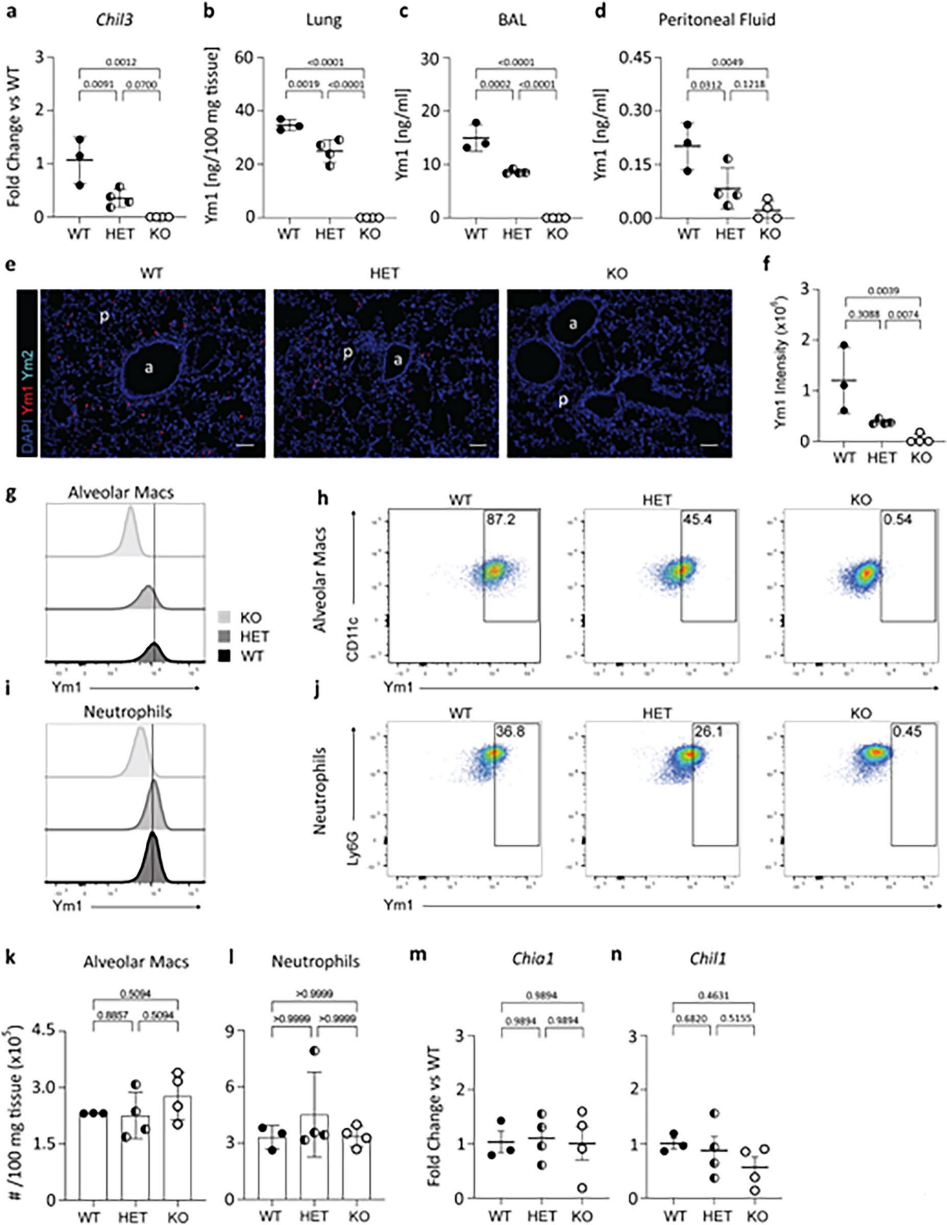
Phenotyping of the backcrossed BALB/c *Chil3*-deficient mice. **a** *Chil3* mRNA expression in wildtype (WT), heterozygous (HET), and knock out (KO) mice from whole lung tissue. **b** *Chil3* encodes the protein Ym1 which was quantified in whole lung homogenate normalised to the mass of the lung, **c** the bronchoalveolar lavage, and **d** peritoneal lavage from WT, HET, and KO Ym1*-*deficient mice. **e** Immunofluorescence showing the expression of Ym1 and Ym2 in lung tissue from naïve mice in WT, HET, and KO *Chil3* deficient mice. **f** Quantification of the fluorescent intensity of Ym1 in WT, HET and KO Ym1-deficient mice. Plots showing the MFI of Ym1 staining in alveolar macrophages (**g** and** h**) and neutrophils (**i** and** j**) from WT, HET, and KO Ym1-deficient lungs. **k** Total number of alveolar macrophages, and **l** neutrophils, identified from flow cytometry from digested whole lung tissue. RNA expression of active chitinase *Chia1* (**m**) and CLP *Chil1* (**n**) from whole lung in WT, HET, and KO Ym1-deficient mice. For qPCR mRNAs were normalised to levels in WT mice and are relative to the mean of housekeeping genes *Rn18s* and *Rpl13a*. Annotations in e) show airways (a) and parenchyma (p). Data were analysed by ANOVA with Tukey’s multiple comparison test with significance level showing comparisons between each different genotype, p values are enumerated within each panel. CLP = chitinase-like protein

Ym1 protein, encoded by the *Chil3* gene*,* is abundant in the steady state murine lung and known to be expressed by neutrophils and alveolar macrophages (Nio et al. 2004). Therefore, we initially validated the loss of Ym1 in lung tissue from fully backcrossed BALB/c Ym1-deficient mice. Expression of *Chil3* was quantified by reverse transcription quantitative PCR in lungs from naïve mice. *Chil3* was readily detectable in wildtype animals but completely absent in homozygous mice, with intermediate expression in heterozygous littermates (Fig. 6a). This pattern was also observed at a protein level as quantified by enzyme-linked immunosorbent assay (ELISA) in both whole lung tissue homogenate (Fig. 6b) and the bronchoalveolar lavage (BAL) (Fig. 6c). Ym1 protein levels were similarly reduced in peritoneal fluid although, as expected, Ym1 concentration in the peritoneal cavity was much lower than lung or BAL in the steady state (Fig. 6d).

To characterise the pulmonary cellular expression pattern of Ym1 and to validate ELISA results immunofluorescence staining in lung sections was performed. Ym1^+^ cells were found throughout the parenchyma of wild-type lungs, consistent with its expression in alveolar macrophages (Ward et al. 2001; Nio et al. 2004) (Fig. 6e). Sections from Ym1-deficient mice showed no positive staining for Ym1 with heterozygote animals again demonstrating an intermediate phenotype (Fig. 6e) around half that of the wildtype (Fig. 6f). To confirm these quantitative differences the mean fluorescent intensity (MFI) of Ym1 was quantified by flow cytometry on digested lung single cell suspensions. A reduction in Ym1 MFI from wildtype to knockout animals was observed in histograms of lung alveolar macrophages (Fig. 6g and h) and lung neutrophils (Fig. 6i and j). However, there was no difference in pulmonary alveolar macrophage (Fig. 6k) or neutrophil (Fig. 6l) numbers between genotypes.

Due to the similarity between the GH18 family (Fig. 1) we wanted to ensure that expression of other chitinases and CLPs were not altered in our Ym1-deficient mice. *Chil4* (Ym2) is not highly expressed in the naïve lung and did not increase in the *Chil3* deficient mice (data not shown). However, both *Chil1* (BRP-39) and *Chia* (AMCase) are detectable in naïve lung tissue (Sutherland et al. 2014). Notably *Chia* is located immediately upstream of *Chil3* and would be potentially susceptible to alterations from the targeting of *Chil3*. Reassuringly, there were no differences in expression of *Chil1* or *Chia* between any of the genotypes (Fig. 6m and n). Together, our findings demonstrate CRISPR-mediated deletion of Ym1 with no effect on the expression of the associated chitinases or CLPs in the steady state. Considering how little is known about the function of Ym1, this strain will facilitate novel understanding of how this widely expressed protein functions in health and disease.

## Discussion

The C57BL/6 inbred strain of mice was originally established in the 1920s and has been a well characterised model in many areas of research including cancer and immunology (Song and Hwang 2017). C57BL/6 was selected by the Mouse Genome Sequencing Consortium as the reference genome for the laboratory mouse (Chinwalla et al. 2002), the second mammalian genome sequenced after humans. Subsequently it was also chosen as the background strain for the Knockout Mouse Project (Ringwald et al. 2011). This widespread use means that assisted reproduction techniques are well established, facilitating the direct culture and genetic manipulation of C57BL/6 embryos. However, the use of C57BL/6 is not without its limitations. Generations of inbreeding has inevitably led to genetic drift and strain differences between laboratories and suppliers. Notably, genetic duplications have been identified previously, including a copy number variant that disrupts the function of *Dock2* with implications on immune regulation (Mahajan et al. 2016). Other genetic loss of function variants identified in C57BL6 include *Nnt* (Freeman et al. 2006), *Mnrn1* (Kumar et al. 2013), and *Rd8* (Mattapallil et al. 2012)*.* In this study we attempted to create a conditional *Chil3* mouse model on the C57BL/6 background using a CRISPR-Cas9 system. However, co-segregation of different gene edits, followed by ddPCR based copy number analysis, indicated a duplication of the CLP locus in the supplied mice. This aligned with previous data indicating a duplication of this locus in C57BL/6 strains (Graubert et al. 2007), later confirmed by others (Zhu et al. 2020).

Gene duplication events are considered to be one of the major drivers of genetic evolution (Ohno 2013), allowing for the development of new gene functions from existing genetic code. The CLP locus in particular is a hotspot for gene duplication events and the generation of *Chil3* and *Chil4* in the murine genome was due to such as event (Okawa et al. 2023). This duplication event, as well as promoter polymorphisms, have also been shown to correlate with increased expression of Ym1 protein across inbred strains (Zhu et al. 2020). Related to this study, we have also generated a *Chil4* “knockdown” mouse on the C57BL/6 background (Parkinson et al. 2023) in which one copy of the duplicated *Chil4* gene was deleted. This corresponded with the loss of secreted Ym2 into the airways during allergic airway inflammation. However, Ym2 expression within epithelial cells was maintained, suggesting differences in the localisation of the proteins encoded by the different copies of *Chil4*. Considering the wide adoption of the C57BL/6 J mouse in comparative studies (Sellers et al. 2012; Walkin et al. 2013; Iwasaki 2016) it will be important to understand the biological implications of these duplications. Inbred mouse strains are a common research tool, yet widespread genetic difference between substrains and also within strains are becoming apparent. It is critical specific mouse substrains used in experiments are well documented, in line with recent Laboratory Animal Genetic Reporting (LAG-R) framework (Teboul et al. 2024) to allow accurate interpretation of results.

The BALB/c strain is an alternative inbred mouse model, also used extensively for immunological research. After confirming the presence of a single copy of the *Chil3* gene in BALB/c mice, we reasoned this would serve as a suitable background for generating a *Chil3*-deficient strain. We devised a whole gene deletion strategy exploiting the non-conserved gene flanking sequences. However, BALB/c embryos proved difficult to generate through superovulation and natural matings. Notably, this difficulty has been reported by several respondents to discussion on the International Society for Transgenic Technologies (ISTT) forum. Hence, embryos generated from F1 hybrids of C57BL/6 and BALB/c mice (CB6) were used instead. We reasoned that this approach would provide a targetable single copy BALB/c allele in embryos, while potentially retaining the manipulation compatibility of C57BL/6 embryos. Whilst creating a *Chil3* knockout allele using the CB6 strategy was successful, the birth rates from embryos injected were still very low (1.7%). One alternative would have been to utilise conventional homologous repair in BALB/c mouse embryonic stem cells, reviewed recently here (Clark et al. 2020). However, recently strategies have emerged that may allow the genetic manipulation of embryos in situ (Imai et al. 2022). This would circumvent the technical difficulty of harvesting and culturing embryos in challenging strains such as BALB/c.

Using CB6 as a background strain has the advantage of allowing faster backcrossing to either of the constituent strains. In this study we initially backcrossed the founder *Chil3*-deletion strain to the BALB/c^olahsd^ strain. Backcrossing was accelerated using a commercial ‘genetic monitoring’ service from Transnetyx. This speed congenic system uses a panel of SNP markers to allow quantification of the contribution of constituent genetic backgrounds. Pups were assessed for heterozygosity allowing determination of the optimal offspring to use for each successive crossing. This approach also allowed assessment of a suitable endpoint to confirm the strain was sufficiently “backcrossed”. Here we have reported data from just the BALB/c backcrossing, but in parallel we have also backcrossed the *Chil3* knock out allele on to a C57BL/6 background. This will be vital for inferring differences in CLP function strains.

Recent work identified a subpopulation of Ym1^+^, common monocyte progenitor-derived, circulating monocytes (Ikeda et al. 2023) that increase in the gut during a model of DSS-induced colitis with increased expression of Ym1. Deletion of these monocytes using a *Chil3*-driven diphtheria toxin receptor model resulted in delayed recovery (Ikeda et al. 2018). It is important to note that it is impossible to disentangle whether delayed recovery was specifically due to reduced Ym1 levels, or the loss of infiltrating monocytes. It should be noted that duplication of the CLP locus had not been published at this point. It would be interesting to evaluate whether the CLP duplication is present in the reporter or DTR strains, and which *Chil3* copies were targeted. Ym1 has long been known to crystallise at high concentrations and pH, particularly in the lungs of aged C57BL/6 J and Sv/129 mice (Guo et al. 2000). Crystalline Ym1 is a far more potent stimuli of type-2 inflammation than the soluble form of the protein (Heyndrickx et al. 2023). Considering the increased concentrations of Ym1 in the serum of strains harbouring the duplication (Zhu et al. 2020) it could be hypothesised that this is one factor predisposing C57BL/6 mice to crystal formation.

Knockout mouse models for Chitotriosidase (*Chit1*), AMCase (*Chia*) and Brp39 (*Chil1*) already exist (Skarnes et al. 2011; Nikota et al. 2011), and have highlighted a role for these molecules in several conditions. *Chia* is highly expressed in the murine lung and *Chia*-deficient mice demonstrate a reduced type-2 immune response after infection with the helminths *Nippostrongylus brasiliensis* and *Heligmosoides polygyrus*. Despite this they induce equivalent allergic airway inflammation in spite of reduced numbers of group 2 innate lymphoid cells (Vannella et al. 2016). The chitin degrading activity of AMCase also appears to be important for limiting age-related fibrosis (Van Dyken et al. 2017). Conversely *Chit1* is highly expressed in the human lung (Seibold et al. 2008) but has also been shown to be important in the development of bleomycin-induced lung fibrosis and is upregulated in human idiopathic pulmonary fibrosis (Sklepkiewicz et al. 2022). Similarly, *Chil1* was shown to be upregulated in cigarette smoke-induced lung damage. However, *Chil1* itself was redundant for the induction of inflammation in this model (Nikota et al. 2011). An emerging concept in the CLP field is one of compensation and redundancy between the CLPs in different settings. Previous papers have shown that overexpression of YKL-40 in the mouse lung can rescue the defective Th2 response (Cg et al. 2009) and exacerbated hyperoxic acute lung damage (Sohn et al. 2010) observed in Brp39-deficient mice. Whilst there is no direct human orthologue of Ym1 it functionally corresponds well to human YKL-40. Both Ym1 and YKL-40 are expressed in neutrophils and macrophages and are increased in a wide range of inflammatory conditions. Therefore, understanding the fundamental functions of Ym1, and other murine CLPs, in health and disease will shed mechanistic light on the many roles of CLPs in humans. Notably, whether these proteins are useful biomarkers and if they could be potential therapeutic targets. Future research should aim to explore the roles of all the murine CLPs (Brp39, Ym1, and Ym2) and correlate these with human data in order to make robust translational hypotheses.

## Materials and methods

### Animals

BALB/c^OlaHsd^ and C57BL/6J^OlaHsd^ mice were purchased from (Envigo, Hillcrest, UK), housed in individually ventilated cages within specific pathogen-free conditions at the University of Manchester Biological Services Facility. All animal procedures were performed in accordance with the UK Animals (Scientific Procedures) Act of 1986 under Project License (70/8548 and P4115856) granted by the UK Home Office and approved by the University of Manchester Animal Welfare and Ethical Review Body. Euthanasia was performed by asphyxiation in a rising concentration of CO_2_ followed by confirmation of death by cessation of circulation or cervical dislocation.

### In silico protein analysis

The biochemical properties of the chitinases and chitinase-like proteins was extracted from the Uniprot “align” function (https://www.uniprot.org/align). These data were then combined and imported into R for graphical representation.

### CRISPR-Cas9 reagents

For the attempted conditional allele, we designed sgRNA specific to the introns flanking exon 3 for Chil3 that had low off target potential using https://wge.stemcell.sanger.ac.uk/. The 5’ sgRNA *ctgatattcttattctaccc* and 3’ sgRNA *atatggggtcgtataacatg* were subcloned into pUC57-sgRNA expression vector (Addgene 51132 (Shen et al. 2014)). Vectors were linearised and used as a template for sgRNA synthesis using HiScribe (NEB) and RNA purified using Ambion MEGAclear transcription clean up kit, all according to manufacturer’s instructions. A dsDNA repair template comprising homology to the region flanking LoxP-exon3-loxP sequences was cloned. For Chil3 gene deletion we designed two sgRNA targeting intergenic regions up- and down-stream of the gene (upstream sgRNA *ggggccttctcctaaagatt,* and *attcccaaatctttaggaga,* downstream *tactgtccacctcgagtggt*, and *actgtccacctcgagtggtg*), and ssODN repair template *tgccatctctggggacacacagtggccttccacaggagattagattcccaaatctttaggactcgaggtggacagtacaatttcaccctctccactgtagagcacttcatcagagggcta* to homologously repair across excised DNA. These reagents were all purchased as Alt-R products (Integrated DNA Technologies, Coralville, USA).

All editing reagents were purified or resuspended in sterile, RNase free Injection buffer (TrisHCl 1 mM, pH 7.5, EDTA 0.1 mM). For microinjection sgRNA were used at 20 ng/ml, EnGen Cas9 protein (NEB) 20 ng/ml and repair templates 10 ng/ml and 50 ng/ml respectively for dsDNA or ssODN DNA. Injection mixes were microinjected into one-day single cell mouse embryos from respective strains, generated through overnight matings following superovulation (Quadros et al. 2017; Behringer et al. 2014). Zygotes were cultured embryos surgically implanted into the oviduct of day 0.5 post-coitum pseudopregnant mice. After birth and weaning, genomic DNA was extracted using Sigma REDExtract-N-Amp Tissue PCR kit and used to genotype pups. For the LoxP insertions we used flanking PCRs and sequencing to identify potentially positive pups, followed by re-amplification of products with high fidelity KOD polymerase, and subsequent blunt cloning into pCR-Blunt (Invitrogen). 12 colonies of transformed and miniprepped plasmid were Sanger sequenced using the M13R primer, and three different reads detected as indicated in Fig. 2d-e. We also integrated unique primer sequences in addition to LoxP sites to use for genotyping. Primer sequences are below, and position annotated in Fig. 2. For the gene deletion Primers immediately up and down stream of the sgRNA we used in combination. On an unedited allele no product would be generated, on a deleted allele a 384 bp product is created, and was sequence confirmed. Primer sequences are below, and position annotated in Fig. 4. HDR mediated gene deletion was confirmed by Sanger sequencing.

### Droplet digital PCR

We used droplet digital PCR (ddPCR) to confirm duplication in our mouse strains. ddPCR was conducted using the QX200 Droplet Digital PCR System (BioRad). Primers and probes were generated to respective CLPs (Table S1). Samples were generated as recommended by the manufacturer using the ddPCR Supermix for Probes (No dUTP) (186–3023), 900 mM forward and reverse primers alongside 250 mM hydrolysis probes. Oil emulsion was generated using the QX200 Droplet Generator and Droplet Generation Oil for Probes (1,863,005), DG8 Gaskets (1,863,009) and DG8 Cartridges (1,864,008). Oil emulsions were then loaded onto ddPCR™ 96-Well Plates (12,001,925) and sealed with a PCR Plate Foil Heat Seal (1,814,045) using a PX1 PCR Plate Sealer (1,814,000). PCR products were then amplified using a C1000 Touch Thermalcycler (BioRad) using manufacturer suggested settings and an annealing temperature of 60 °C (Table 4). Amplified droplets were then measured using the QX200 Droplet Reader and results analysed using QuantaSoftTM Analysis Pro to determine the copy number of the specific genes of interest in relation to a control gene *Tfrc* (Transferrin Receptor).

### Speed congenic backcrossing

Genetic background testing was performed using the genetic monitoring service from Transnetyx. Ear punches were collected and shipped to Transnetyx for initial genotyping of the *Chil3* deletion allele (assay available from Transetyx). Heterozygous mice were then submitted for genetic background testing using the miniMUGA panel (Neogen). > 10,000 diagnostic single nucleotide polymorphisms are assessed to quantify the background of the assessed mouse. Animals with the highest levels of homozygosity across the whole genome were used for the next round of backcrossing.

### Cell preparation

The left lung lobe was placed in 10% (v/v) neutral-buffered formalin (NBF) (Sigma-Aldrich) for 24 h before being transferred for storage in 70% (v/v) ethanol (Fisher Scientific). Subsequently lungs were processed using an alcohol series and paraffin embedded. Sections of 5 μm were cut using a microtome and mounted on slides (Superfrost Plus Adhesion). For immunofluorescence staining of lung tissue, sections were de-waxed through xylene and rehydrated through a series of alcohols before heat-induced antigen retrieval using 10 mM sodium citrate buffer, at pH 6.0. Samples were then blocked for nonspecific binding using 10% (v/v) donkey serum in PBS (Sigma-Aldrich) followed by avidin biotin blocking (BioLegend). Primary antibodies were added to samples (Table 5) and incubated overnight at 4 °C. Slides were washed in PBS before application of secondary antibodies (Table 5) for incubation at room temperature for 2 h. Sections were mounted using coverslips and Fluormount G (Southern Biotech) containing 4ʹ,6-diamidino-2-phenylindole (DAPI). Images of staining were captured using an EVOS FL imaging system (Thermo Fisher Scientific). To calculate Ym1 staining intensity, regions of interest (ROIs) were drawn around airways and overlaid into the images using ImageJ software (version 1.51 s). An ImageJ macro was used, with manual background threshold determined by a negative region, to calculate the integrated staining density across each ROI. Data was exported for graphing in GraphPad Prism (version 10.2.3).

**Table 5:** ddPCR primers and probes

Name	Sequence	Tag
*Chil3* F	GGGATGATATAGAGGTTTAGG	
*Chil3* R	TATTGGCATCTGGTCTTG	
*Chil3* Probe	ATGGGAAACTAGCAGTAGCCACCA	FAM
*Chil4* F	GAATCTACTGATTCCCATCTC	
*Chil4* R	CAGCCATTCATTAACTTACAC	
*Chil4* Probe	TTCAGTAAGGAGGCACAGGAAGAG	FAM
*Chia* F	CATGATCTGGGCCATTG	
*Chia* R	AGGGCTTTGTTCAAAGTAG	
*Chia* Probe	ACTGGCTCTTTCTGTGATCAGGGA	FAM
*Chil1* F	TTCCTGCGTTCTTATGG	
*Chil1* R	GATCAGGGTGGAGAAATAC	
*Chil1* Probe	CCTGGCTCTACCCTCGCTTAAGAG	FAM
*Tfrc* F	CTGGTCAGCTCATTATTAAAC	
*Tfrc* R	GAACTGGTTCAGATCCTTC	
*Tfrc* Probe	ACCCATGACGTTGAATTGAACCTGG	HEX
*Dot1l* F	TAGTTGGCATCCTTATGCTTCATC	
*Dot1l* R	GCCCCAGCACGACCATT	
*Dot1l* Probe	CGACTTGAGAGCTGG	FAM

### Cell preparation

Bronchoalveolar lavage (BAL) was collected via cannulation of the trachea and washing of the airways four times with 0.4 mL PBS (Sigma-Aldrich). Samples were centrifuged (400 xg) and supernatant collected and stored at -80 °C. Superior and inferior lung lobes from the right side of the mouse were removed and chopped finely using blunt nosed scissors prior to the addition of digestion mixture containing 0.2 U/mL Liberase TL (Sigma-Aldrich) and 80 U/mL DNase Type 1 (Invitrogen) in pre-warmed RPMI (Gibco). Digestion was carried out for 45 min in a shaking incubator (220 rpm) at 37 °C. Digested tissue was mashed through a 70 μm cell strainer (Greiner Bio- One) and red blood cells were lysed using ammonium-potassium-chloride (ACK) lysis buffer (Invitrogen). Single cell suspensions of digested lung tissue were then counted to acquire total live cell numbers using ViaStain AOPI staining solution (Nexcelom Bioscience) and a Cellometer Auto 2000 cell counter (Nexcelom Bioscience) before plating cells to stain for flow cytometry.

### Flow cytometry

For surface staining, approximately equal numbers of cells per lung sample were washed in PBS prior to staining with Live/Dead (1:1000) (Thermo Fisher Scientific) and incubated with Fc-block (1:200) (CD16/CD32; clone 2.4G2; BD Biosciences) for 15 min. Antibodies (Table 6) were added in PBS containing 2 mM EDTA and 1% (v/v) FBS. For analysis of cytokines cells were stimulated for 4 h at 37 °C with a cell stimulation cocktail containing protein transport inhibitor (1:500) (eBioscience). For intracellular staining, cells were fixed with fixation/permeabilization buffer (Invitrogen) before addition of permeabilisation buffer (Invitrogen). Cells were acquired on an LSRFortessa flow cytometer (BD Biosciences) using FACSDiva software (BD Biosciences) and analysed using FlowJo v10 software (BD Biosciences).

**Table 6:** Antibodies used for immunofluorescence staining

Antigen	Antibody clone	Dilution	Manufacturer
Ym1	Goat polyclonal – biotinylated	1:100	R&D (BAF2446)
Ym2	Rabbit polyclonal	1:1000	Generated in-house
Goat IgG	Donkey anti-rabbit IgG 637	1:200	R&D (NL005)
Biotin	Streptavidin 557	1:800	R&D (NL999)

### qRT-PCR

The post-caval lung lobe was collected in RNA Later solution (Thermo Fisher Scientific) and stored at -80 °C before RNA extraction using the PureLink RNA Mini kit (Invitrogen) according to manufacturer’s instructions. RNA yield and purity were determined using a NanoDropTM. RNA (500 ng) was reverse transcribed using 5 × RT buffer (Bioline), Tetro reverse transcriptase (Meridian Bioscience), oligo dT 15-mer (Integrated DNA Technologies), Ribonuclease inhibitor (RNasin) and 10 mM deoxynucleoside triphosphates (Promega). Quantitative real-time PCR was performed using SYBR green master mix (Agilent Technologies), specific primer sets (Table 7, Integrated DNA Technologies), and read on a LightCycler 480 II (Roche). Changes in gene expression levels were determined using the ∆∆Cq method relative to the geometric mean of housekeeping genes RpI13a and Rn18s.

**Table 7:** Antibodies used in Flow cytometry

Antigen	Fluorophore	Clone	Dilution	Manufacturer
CD11c	APC-Cy7	N418	1:1000	BioLegend
CD11b	BV711	M1/70	1:1000	BioLegend
CD45	BV785	30-F11	1:400	BioLegend
CD64	BV605	X54-5/7.1	1:400	BioLegend
Siglec-F	BV421	E50-2440	1:400	BD Biosciences
Ly6G	AF647	1A8	1:400	BioLegend
MHCII (I-A/I-E)	BV650	M5/114.15.2	1:1000	BioLegend
Ym1*	PE-Cy7	Polyclonal	1:200	Novus

^*^Biotinylated Ym1 used at 1:200 and detected using streptavidin (1:400)

### Protein quantification

The right middle lung lobe was snap frozen on dry ice when collected and then homogenised in a set volume of PBS at 20 Hz using a Tissue Lyser II (Qiagen). Lung homogenates were centrifuged at 16,000 xg for 5 min, supernatants were then collected and stored at -80 °C prior to protein analysis. Levels of Ym1 were quantified by ELISA (DuoSet) as per manufacturer’s instructions with all samples measured undiluted. Concentrations were normalised to the weight of lung tissue with homogenate volume accounted for in calculations (Table 8).

**Table 8:** Primer sequences for detection of mRNA of listed genes by quantitative reverse transcription PCR

Gene	Forward Primer	Reverse Primer
Chil3	ACCTGCCCCGTTCAGTGCCAT	CCTTGGAATGTCTTTCTCCACAG
Chil4	CAGAACCGTCAGACATTCATTA	ATGGTCCTTCCAGTAGGTAATA
Chil1	AGCAGTATTTCTCCACCCTGAT	ACCTTTCCTGCTGACAAAGC
Chia1	GTCTGGCTCTTCTGCTGAATGC	TCCATCAAACCCATACTGACGC
RpI13a	CATGAGGTCGGGTGGAAGTA	GCCTGTTTCCGTAACCTCAA
Rn18s	GTAACCCGTTGAACCCCATT	CCATCCAATCGGTAGTAGCG

### Statistical analysis

GraphPad Prism (version 10.2.3) was used for statistical analysis. Normality of data was assessed using the Shapiro–Wilk test with some datasets log2 transformed to achieve normal distribution. For normally distributed data parametric tests were used and for non-normally distributed data, non-parametric tests were used. For comparisons between multiple groups, one-way analysis of variance (parametric) with Holm-Sidak’s multiple comparisons test or Kruskal–Wallis test (non-parametric) with Dunn’s multiple comparison test were used, as indicated in figure legends. Data are represented as mean ± SD and exact P values are shown on graphs.

## Supplementary Information

Below is the link to the electronic supplementary material.

Supplementary file1 (DOCX 832 kb)

## Declarations

### Data Availability

All data is available in the manuscript or upon request.
