# Supplementary material for: Generation of a Ym1 deficient mouse utilising CRISPR-Cas9 in CB6 embryos
Source: Transgenic Res. 2025 Sep 25;34(1):44. doi: 10.1007/s11248-025-00455-8 (PMC12464116; doi:10.1007/s11248-025-00455-8)
Supplement: Supplementary file 1 — Supplementary file1 (DOCX 832 kb) [file 11248_2025_455_MOESM1_ESM.docx]

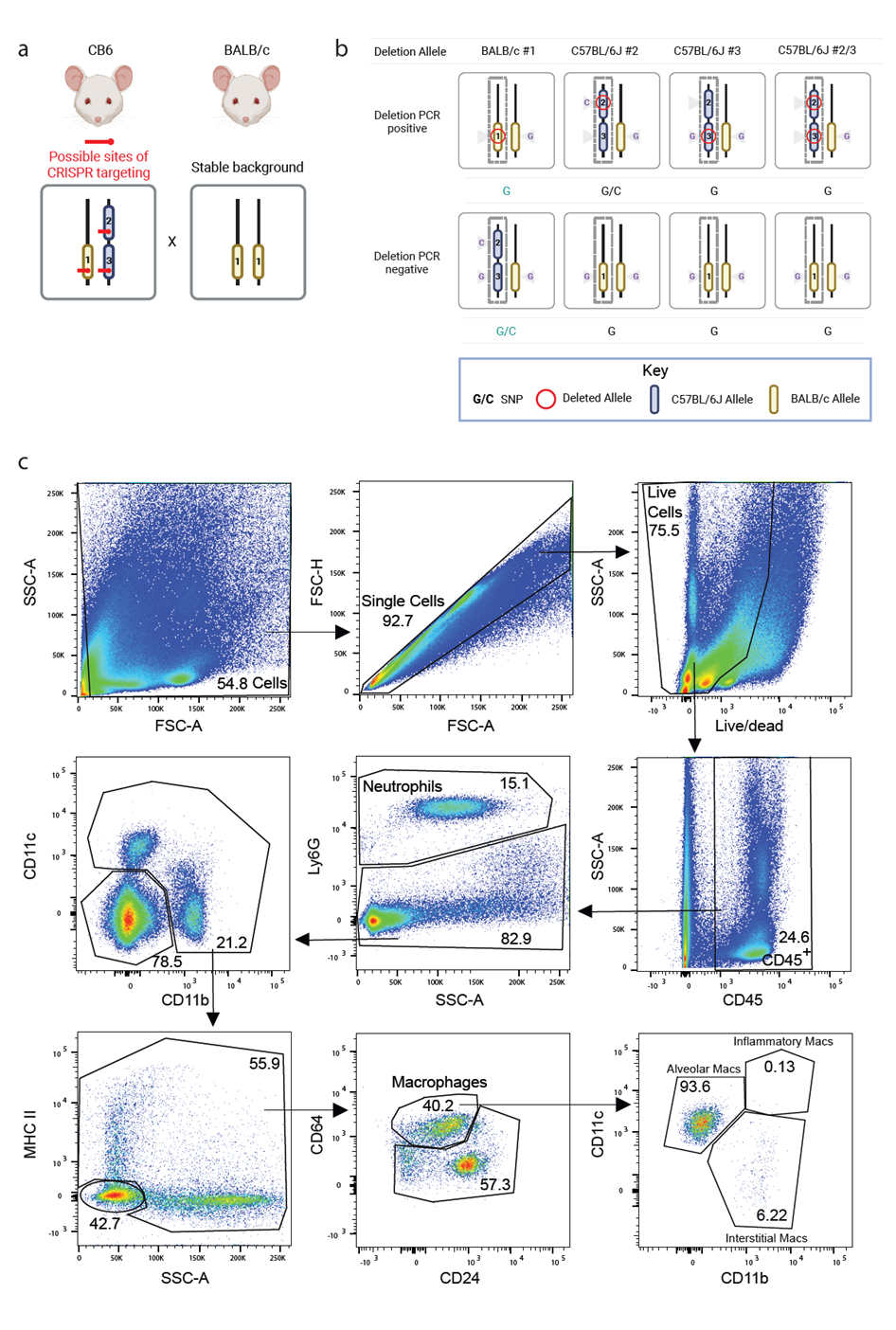
Supplemental Data

**Supplemental Data**: The presence of specific SNPs in the region described in Figure 4 allows for assessment of which CLP locus had been targeted. **a**) Initial crossing of targeted CB6 mouse to a wildtype BALB/c animal. **b**) Schematic showing the possibly results of the identified SNP for genotyping the F1 mice their correlation with the allele which had been deleted. **c**) Flow cytometry gating strategy applied to cells from digested lung tissue. Cells were selected using size (FSC-A) and granularity (SSC-A) prior to exclusion of doublets (FSC-H and FSC-A) and dead cells (Live/Dead-). CD45^+^ cells were then selected prior to separation of immune cell populations. Neutrophils were identified as Ly6G^+^. Alveolar macrophages were identified as Ly6G^-^CD11c^+^SSC-A^hi^CD64^+^CD11b^-^. (FSC-A, forward scatter area; SSC-A, side scatter area; FSC-H, forward scatter height).
